# The computational age‐at‐death estimation from 3D surface models of the adult pubic symphysis using data mining methods

**DOI:** 10.1038/s41598-022-13983-8

**Published:** 2022-06-20

**Authors:** Anežka Kotěrová, Michal Štepanovský, Zdeněk Buk, Jaroslav Brůžek, Nawaporn Techataweewan, Jana Velemínská

**Affiliations:** 1 Department of Anthropology and Human Genetics, Faculty of Science, Charles University, Vinicna 7, Prague 2, 128 43 Czech Republic; 2 Faculty of Information Technology, Czech Technical University in Prague, Thakurova 9, Prague, 160 00 Czech Republic; 3 Department of Anatomy, Faculty of Medicine, Khon Kaen University, Khon Kaen, Thailand

**Keywords:** Computational models, Machine learning, Skeleton, Data mining, Bone

## Abstract

Age-at-death estimation of adult skeletal remains is a key part of biological profile estimation, yet it remains problematic for several reasons. One of them may be the subjective nature of the evaluation of age-related changes, or the fact that the human eye is unable to detect all the relevant surface changes. We have several aims: (1) to validate already existing computer models for age estimation; (2) to propose our own expert system based on computational approaches to eliminate the factor of subjectivity and to use the full potential of surface changes on an articulation area; and (3) to determine what age range the pubic symphysis is useful for age estimation. A sample of 483 3D representations of the pubic symphyseal surfaces from the *ossa coxae* of adult individuals coming from four European (two from Portugal, one from Switzerland and Greece) and one Asian (Thailand) identified skeletal collections was used. A validation of published algorithms showed very high error in our dataset—the Mean Absolute Error (MAE) ranged from 16.2 and 25.1 years. Two completely new approaches were proposed in this paper: SASS (Simple Automated Symphyseal Surface-based) and AANNESS (Advanced Automated Neural Network-grounded Extended Symphyseal Surface-based), whose MAE values are 11.7 and 10.6 years, respectively. Lastly, it was demonstrated that our models could estimate the age-at-death using the pubic symphysis over the entire adult age range. The proposed models offer objective age estimates with low estimation error (compared to traditional visual methods) and are able to estimate age using the pubic symphysis across the entire adult age range.

## Introduction

Age‐at‐death estimation is an important indicator in forensic anthropology, especially in the identification of the biological profile of an individual^1^. The current situation with age estimation methods in adults has resulted in broad overlapping or open-ended age intervals, for which they are often criticized^2–5^. Traditional indicators of adult age still face lower classification accuracy, and reliable estimates are only available at three broad age intervals: young adults, mature adults, and the elderly^6–11^. It is also difficult to estimate the age of individuals older than 50–60 years.^12^.

The results of age estimation methodologies on different reference collections have shown a non-linear and inconsistent relationship between the bone metamorphosis of traditional indicators and the chronological age^6^. Some studies indicate that the limiting factor of age estimation can be the visual evaluation of age-related changes^13^.

The new focus of research in forensic anthropology and bioarchaeology is being shifted to sophisticated data mining (machine learning) methods, as the advanced mathematical approaches are continuing to evolve and are more available. These methods are used to discover and extract meaningful information from the provided dataset and to build a model based on that dataset. Together with medical imaging technologies and surface scanning technologies, they are penetrating the methods of the estimation of the biological profile (e.g.^14–17^), and age-at-death estimation is no exception^8,18–20^. Their great potential lies primarily in freeing the evaluator from the subjective visual evaluation of degenerative age-related changes. Secondly, they can find previously unknown age-at-death-related information (features) and numerically quantify it. Thus, they provide repeatable results, which is not guaranteed if a given sample is evaluated by a human. The very first attempts to replace experience-based visual approaches with the use of geometric morphometrics and other advanced mathematical approaches to quantify age-related surface changes date back to the 2010s^21–26^. Attention has been paid mainly to the pubic symphyseal surface^22,23,25^ or the auricular surface^26^. Biwasaka et al. computed mean curvatures (specifically mean curvatures of every 5 mm^2^) of the pubic symphyseal surface and examined the concavo-convex condition of the surface^22^. A mathematical approach analyzing five variables of the curvature variation of the pubic bone symphyseal surface and the auricular surface was proposed by Villa et al.^26^. These variables were: the arithmetic mean of the absolute values of curvature, the highest and the lowest ten percent of the curvature values, and the percent of the convex and of the flat surface (curvature values higher than zero and between − 0.01 and 0.01, respectively). Villa et al. achieved moderate correlations with the decades of the actual age-at-death, similar to those of traditional methods, and concluded that such an approach has potential. Among computational approaches, the one from Stoyanova et al.^24,25^, which is focused on pubic symphyseal 3D surface, is the most prominent and offers researchers the user-friendly forAge software. Three different shape scores and two combinations thereof are computed: SAH-Score proposed by Slice and Algee-Hewitt^23^, bending energy and the ventral curvature. However, their approach has several shortcomings; for example, the approach was proposed only for male individuals, or it is not able to distinguish younger from older individuals whose pubic symphyseal surfaces are both rugged but in different ways (irregular in older individuals). This results in the strong underestimation of not only older individuals^25^ but of the whole sample^27^. Validation studies on different population samples uniformly show low correlation with actual age and higher estimation error^27–30^ than the error in the original study (27–32 years), which was comparable to the error of one of the traditional visual methods^25^. Recently, Bravo Morante et al. presented a new quantitative method based on the bandpass filtering of partial warp bending energy^31^, which is a modification of one of the shape scores (bending energy) applied in a previous study^25^. They also restricted their sample to male individuals^31^.

The aim of this study is threefold: firstly, to validate the algorithms proposed in the study of Stoyanova et al. in a large multi-populational dataset; secondly, to propose our own computational approaches for adult age estimation for both sexes. The first one, namely the SASS (Simple Automated Symphyseal Surface-based) age estimation method, is based on explicit features and allows human interpretation of individual influences, contributing to the estimation of age-at-death. The AANNESS (Advanced Automated Neural Network-grounded Extended Symphyseal Surface-based) age estimation method is similar to the black box (no explicit features) and does not allow human interpretation of individual influences. Lastly, the final aim of this study is to confirm or refute the generally accepted assumption that pubic symphysis could be used for age estimation only in a restricted age range.

## Material

The 483 adult (18–92 years) *ossa coxae* of males and females used in this study came from 374 individuals of one Asian and four European identified osteological collections (104 individuals provided both left and right sides). Two osteological collections originated in Portugal and are housed at the University of Coimbra: the 21^st^ Century Identified Skeletal Collection (CEI/XXI)^32^ and the Coimbra Identified Skeletal Collection (CISC)^33^. The third collection is the Heraklion Collection stored at the facilities of the Forensic Pathology Division of the Hellenic Ministry of Justice and Human Rights in Crete, Greece^34,35^. The fourth European collection is from Switzerland: the Simon Identified Skeletal Collection, housed at the Laboratory of Prehistoric Archaeology and Anthropology of the University of Geneva^36^. The Asian Khon Kaen University Collection (KKU) is stored at the Department of Anatomy in the Faculty of Medicine at this university^37,38^. Ethics approval was granted by the Ethics Committee in Human Research of Khon Kaen University (Reference number HE601315). Table 1 shows the numbers of *ossa coxae* used in our study from each osteological collection and Table 2 presents detailed descriptions of sample age distribution in six age categories, both for each sex separately.

**Table 1:** Summary table of osteological collections and numbers of *ossa coxae* used in the present study for each sex separately.

Collection sex	Portugal 1	Portugal 2	Switzerland	Thailand	Greece	Total
Male	46	84	45	114	10	299
Female	33	58	21	67	5	184
Total	79	142	66	181	15	483

Portugal 1 = The 21st Century Identified Skeletal Collection (CEI/XXI); Portugal 2 = The Coimbra Identified Skeletal Collection (CISC).

**Table 2:** Age distribution of the sample; numbers of pelvic bones in each age category.

	18–29	30–39	40–49	50–59	60–69	70 +	Total
	(years)						
Male	24	54	57	56	59	49	299
Mean age	25.88	34.74	44.95	54.82	64.17	76.80	52.43
Female	9	33	33	39	21	49	184
Mean age	23.78	35.24	45.15	54.26	63.86	80.51	55.81
					Mean age of sample		53.72

## Methods

### Data acquisition

**Figure 1:**
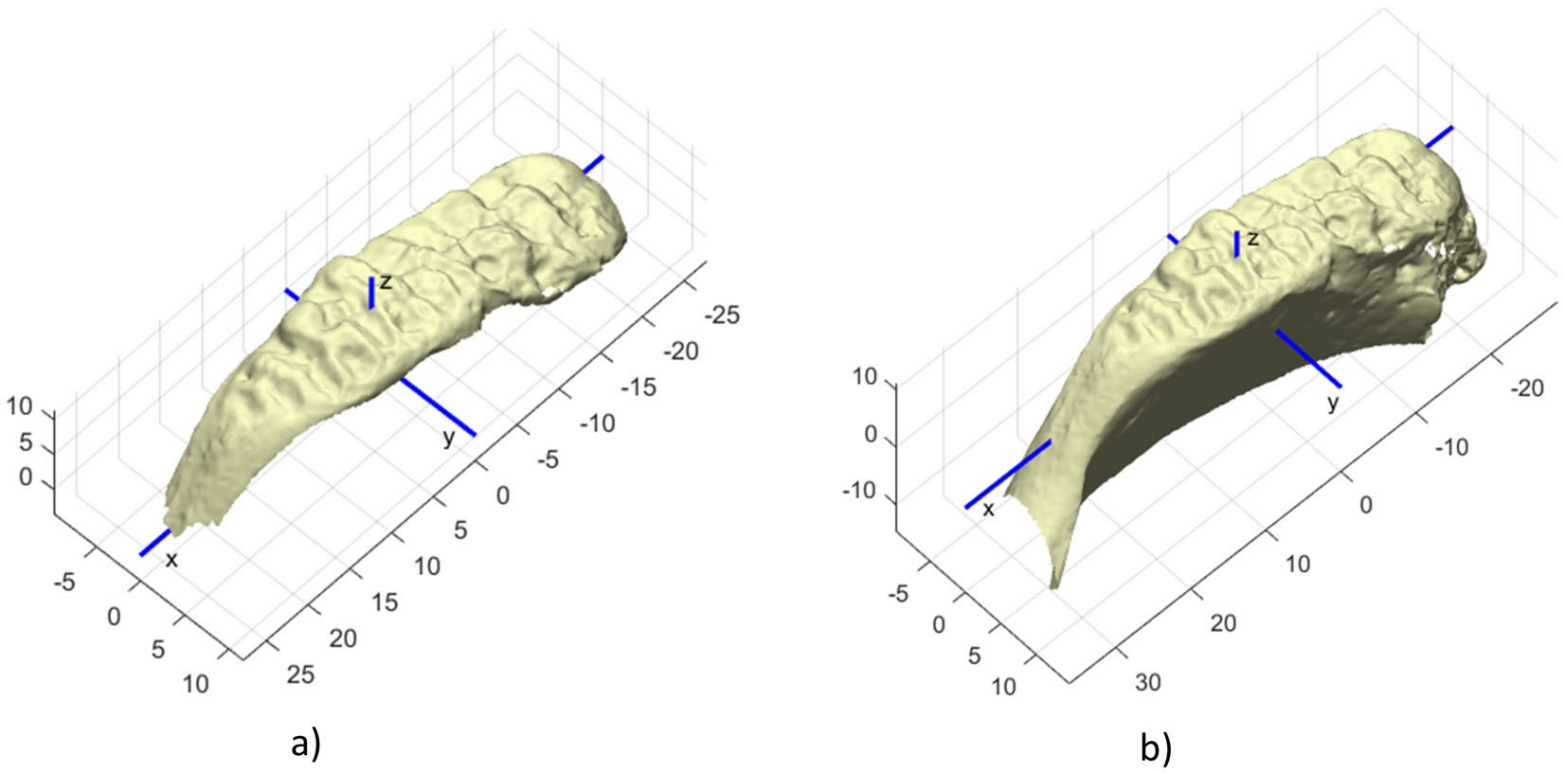
Example of the 3D scan of the pubic symphyseal surface of a 20-year old individual. The Cartesian coordinate system is set in a way that the x-axis defines the dimension with the largest distance/length, the x–y plane projects the articular surface of the bone, so the z-axis defines the variance of the articular surface. (**a**) 3D scan of the articulation surface only; (**b**) 3D scan with an extended area.

All skeletal samples were digitized with the HP 3D Structured Light Scanner PRO S2 or S3 surface scanner and post-processed in the integrated software David LaserScanner v.3.10.4. The whole surface of *os coxae* was scanned but only the pubic symphyseal areas were used. For the first goal (i.e. application of algorithms proposed in the Stoyanova study), only the articulation surface was isolated and exported in PLY (Polygon File) format (see Fig. 1a). For the third goal (i.e. proposing our own approaches), we focused not only on the articulation surface, but also on the pubic symphyseal surfaces with an approximately 1 cm extended area (see Fig. 1b). These were saved in STL (Standard Triangle Language) format. In this format, the surface geometry of a 3D object is represented as numerous small adjacent triangles. When both *ossa coxae* of the same individual were available for our analysis, they were treated individually for validation of Stoyanova et al. models and together in the case of the proposal of our own models. The right-sided isolated pubic symphyseal surfaces were mirrored to the left-side for easier processing. The filling of the holes was performed only for the AANNESS model. The isolation and mirroring were performed in Meshlab software^39^.

### Evaluation of existing algorithms in our dataset

Stoyanova et al. presented in their study five age-estimation models and provided an open source software called forAGE (available at http://morphlab.sc.fsu.edu/). The first model is TPS/BE (Thin Plate Spline algorithm computing Bending Energy), the second is the SAH-Score (Slice and Algee-Hewitt) algorithm^23^, the third is VC (Ventral Curvature), and the last two are a combination of these (VC and SAH-Score, VC and TPS/BE). For detailed information, see the original publications^24,25^. First, we extracted only the articulation surface from all the scans (see e.g. Fig. 1a) and converted it into PLY polygon file format. Then, we used the provided software to evaluate these algorithms in our dataset for each bone individually.

### Our pre-processing methods

The input data, as it comes from the scanner, needs to be pre-processed before applying our age estimation methods. First, we need to standardize the position and orientation of all the scans, as shown in Fig. 1. Although the STL format is a very convenient input data format, the irregular distribution of all the vertices in the 3D space prevents direct processing by the proposed automated age estimation methods (SASS and AANNESS, described below in this section). Therefore, we used two different projections of the surfaces, depending on the automated age estimation method that is then applied.

**Figure 2:**
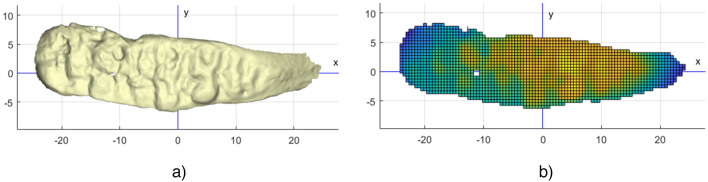
Top view on the articulation surface of the pubic symphysis. (**a**) Irregular triangulation-based surface (triangle edges are not shown); (**b**) articulation surface approximated over regular mesh (individual “pixels” are coloured according to the actual z-value). The actual grid size used for the SASS method is set to 0.1 mm, which is five times smaller compared to this illustration. This provides sufficient resolution for further processing.

In the case of the SASS age estimation method, only the top view of the articulation surface of the pubic symphysis (without extended area) is projected into the x–y plane and approximated over the regular mesh, as is illustrated in Fig. 2.

**Figure 3:**
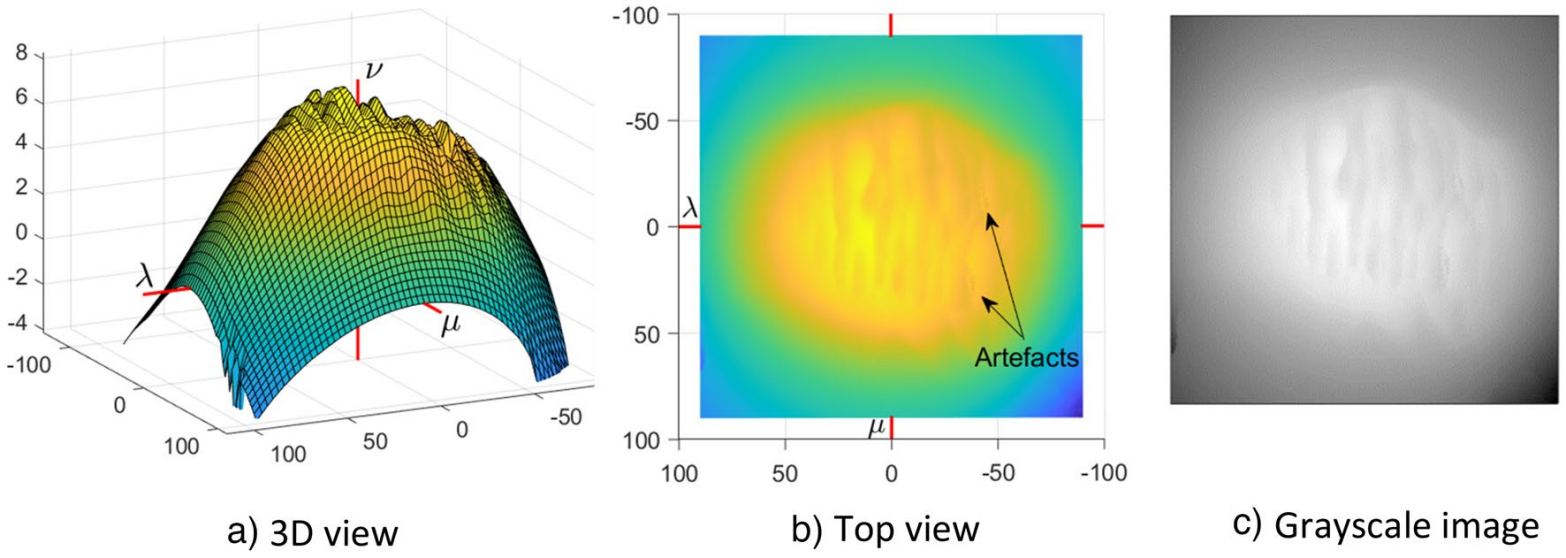
Symphyseal surface from Fig. 1b) in the proposed coordinate system. (**a**) 3D view of the symphyseal surface after the transformation. (**b**) Top view of the symphyseal surface after the transformation. (**c**) The grayscale image generated from the projection of transformed data into the regular mesh.

In the case of the AANNESS age estimation method, the whole surface of the pubic symphysis (including extended area) is transformed into the new coordinate system first, and only then projected onto the plane and approximated over the regular mesh in way that is similar to the SASS age estimation method. This transformation allows us to easily explore not only the articulation surface of the pubic symphysis, but also the extended area covering the side walls of the pubic symphysis. The transformation is described in detail in Buk et al.^40^. Figure 3 shows an example of the symphyseal surface from Fig. 1b in the new coordinate system.

### Proposed age estimation methods

#### SASS age estimation method

**Figure 4:**
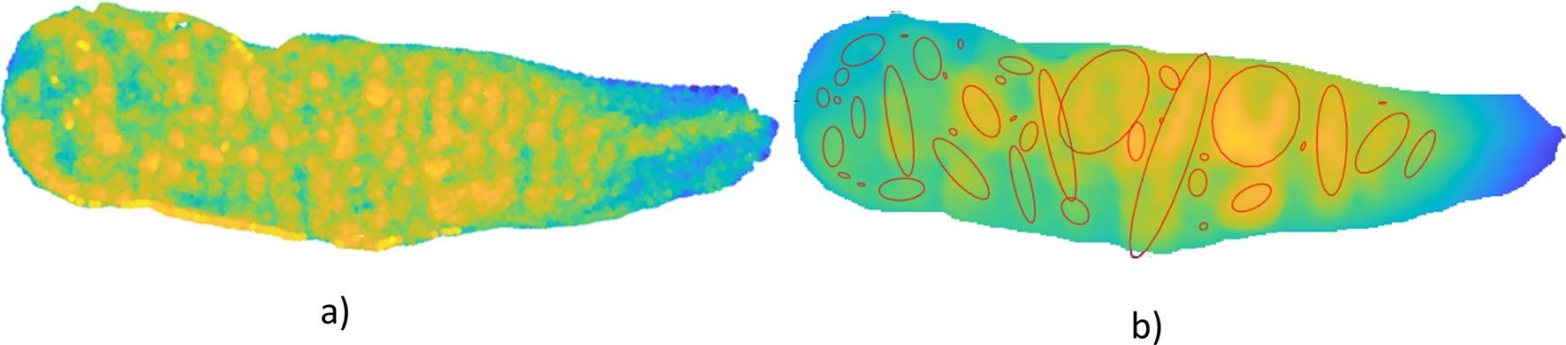
Examples illustrating the feature extraction used in the SASS method. (**a**) Top view of the Dirichlet normal energy distribution over the symphyseal surface. The yellow colour indicates a higher value of the Dirichlet normal energy. The standard deviation of the Dirichlet normal energy computed over the entire surface represents a single feature. (**b**) Detected ellipses over the surface. The yellow colour indicates the highest areas of the scan. The ellipse major axis corresponds to the dominant orientation of the underlying area. The total number of detected ellipses and the number of ellipses with certain orientation (horizontal, vertical) represent other extracted features.

The SASS age estimation method extracts several features from the articulation surface of the pubic symphysis (i.e. without the extended area covering the side walls of the pubic symphysis); based on these features, it estimates the age of the individual by using multi-linear regression. We considered various features, such as the mean surface curvature, mean profile height, the porosity, etc.; however, by using a “greedy” selection algorithm with the Akaike information metric implemented in Weka^41^, we selected the following six features for the SASS method: (1) Dirichlet normal energy (more specifically, standard deviation of Dirichlet local energy values), (2) surface curvature (more specifically, standard deviation of local curvature values), (3) the total number of detected ellipses, (4) the number of horizontally oriented detected ellipses, (5) the number of vertically oriented detected ellipses, and finally, (6) the number of holes in the surface scan. We used the ariaDNE algorithm^42^ implemented in Matlab (available at https://github.com/sshanshans/ariaDNE_code) to evaluate Dirichlet normal energy and surface curvature. Both Dirichlet normal energy and surface curvature were evaluated over the whole articulation surface by using the surface topology (triangular mesh of the articulation surface only and in the resolution as it comes from the 3D scanner). Figure 4a shows an example of Dirichlet normal energy distribution over the symphyseal surface from Fig. 1a). For ellipse detection, we used regular mesh with a grid size of 0.1 × 0.1 mm (see Sect. 3.3, Fig. 2 for instance) and developed our own approach for surface segmentation (based on the local discrepancies of the profile height), allowing us to easily identify disjointed bulging regions. Each such region is approximated with a single ellipse. The ellipse orientation gives us the orientation of the underlying region. Figure 4b) shows an example of detected ellipses over the entire surface. In this case, 39 ellipses are detected in total. The majority of these ellipses are vertically oriented. To decide whether the detected ellipse is considered a horizontal or vertical one, we use not only its orientation, but also the major axis length, the eccentricity, and its size, i.e. omitting all circular-like and very small ellipses. The ellipse is considered to be circular-like if the ellipse eccentricity is less than 0.8. The ellipse is considered to be small if the major axis length is less than 3 mm or if the ellipse area is less than 1.5 mm^2^. Moreover, all ellipses with “uncertain” orientation (about 45°) were also omitted. In this case (shown in Fig. 4b), 13 ellipses are considered as vertically oriented, whereas only 3 ellipses are considered as horizontally oriented. The remaining ellipses (39 − 13 − 3 = 23) are considered circular-like, very small or with uncertain orientation.

The SASS age estimation method uses the following equation to estimate the age-at-death of the individual:

1 $$ {\text{Age}}_{{{\text{estimated}}}} = {\text{a}}_{{1}} {\text{x}}_{{1}} + {\text{a}}_{{2}} {\text{x}}_{{2}} + {\text{a}}_{{3}} {\text{x}}_{{3}} + {\text{a}}_{{4}} {\text{x}}_{{4}} + {\text{a}}_{{5}} {\text{x}}_{{5}} + {\text{a}}_{{6}} {\text{x}}_{{6}} + {\text{K}}, $$

where x_1_, x_2_,… x_6_ are explanatory variables (features), a_1_, a_2_,… a_6_ are slope coefficients corresponding to each explanatory variable, and K is the offset. In our case, x_1_ is the standard deviation of Dirichlet local energy, x_2_ is the standard deviation of local curvature, x_3_ is the total number of detected ellipses, x_4_ is the number of horizontal ellipses, x_5_ is the number of vertical ellipses, and x_6_ is the number of holes in the surface (e.g. due to the bone porosity). As is mentioned above, these features x_1_–x_5_ were selected from a larger set of potential features by the greedy selection algorithm with the Akaike information metric. Therefore, features with no additional benefit or with little age-related information were eliminated. Features x_1_, x_2_ and x_3_ represent the overall surface complexity with no orientation-related information. Although all of these three features represent surface complexity, each captures a different aspect of surface complexity; therefore, their combination contributes to increasing the robustness of the model. Features x_4_ and x_5_ provide additional information about the orientation of the dominant shapes of the surface.

#### AANNESS age estimation method

The AANNESS age estimation method uses not only the articulation surface of the pubic symphysis, but also its side walls, so the surface is extended about 1 cm in each direction. This could potentially provide additional age-related information. The core of the AANNESS age estimation model is a convolutional neural network trained to automatically extract the features from the input images. This contrasts with the SASS method, which uses explicitly defined features.

**Figure 5:**
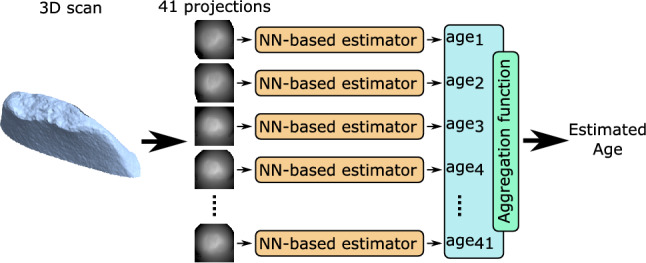
Principle of age estimation for a single individual. There is a single 3D scan for which we build multiple projections (41 in this case). By using the NN-based estimator for each of the projections, we obtain multiple age estimations that are finally aggregated to gain the final estimated age. In this paper, we are presenting the results using the mean aggregation function.

The convolutional part of the neural network is followed by a densely connected feed-forward neural network which is trained to map the features to the age. Both parts are trained together, which is one of the greatest benefits of such a machine-learning approach: we can train the whole system by using the dataset with images (inputs) and ages (outputs). We call this neural network an NN-based (Neural Network-based) estimator—see Fig. 5. When applied to the real bone, we typically use multiple projections of the bone based on its 3D scan. The same NN-based estimator is applied on the particular projection and the results are aggregated to obtain the final age estimation. We have experimented with various aggregation functions like mean, median, mean and median with variance-based outliers removal with no significant difference. In this paper, we are presenting the results using the mean aggregation function. The number of projections is arbitrary—the model is not limited to any specific number. In our experiments, we used 41 projections.

**Figure 6:**
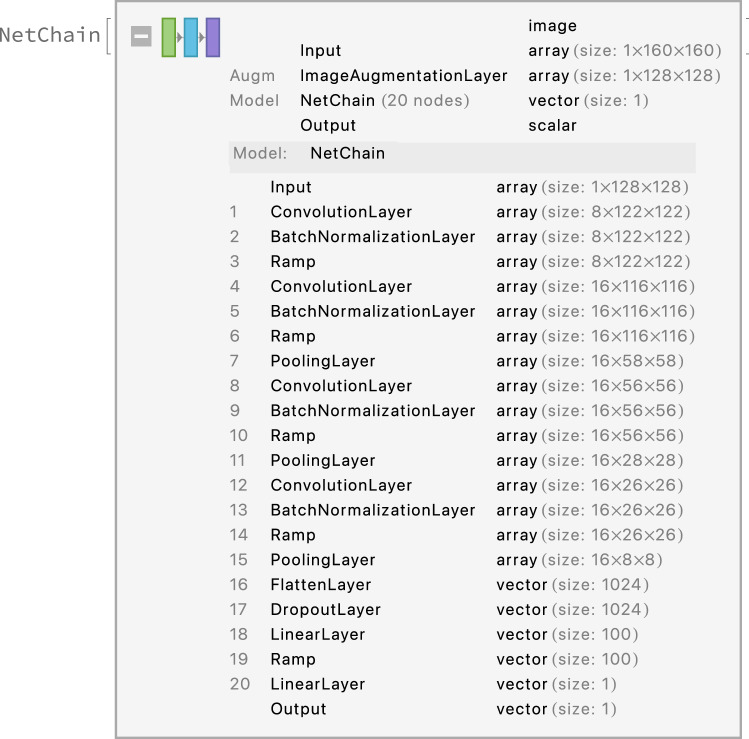
NN-based estimator. The image shows a neural network structure used to estimate age based on the input image. The network consists of multiple layers, where the convolutional layers are mainly responsible for handling the low-level patterns in the image, feature extraction and dimension reduction. The top layers (18–20) are responsible for building high-level relations among the features.

**Figure 7:**
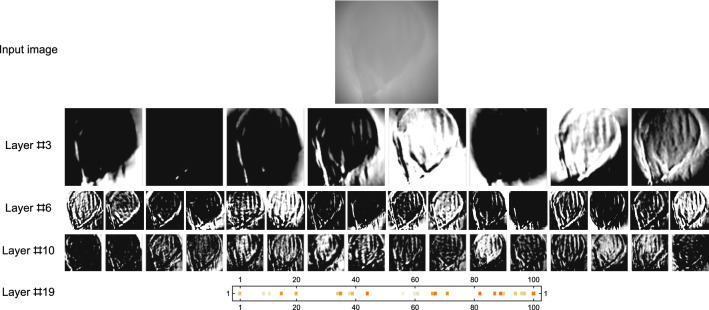
An example of a 72-year-old individual (predicted age = 71.6) evaluation. Even though we can see almost no details in the input image, the model can identify vertical (in this orientation) structures, which seems to be key for age identification.

The structure of the whole estimation process for a single individual is shown in Figs. 5 and 6. Figure 7 shows a visualisation of selected layers in the neural network as a response to an input image. We can see how the network can internally represent the patterns and features. More technical details can be found in the related paper^40^.

### Evaluation of the accuracy of individual models

In order to evaluate the accuracy of a given model and compare it with other models, it is necessary to choose some metrics. For this purpose, we chose Mean Absolute Error (MAE) defined as:

$$MAE=\frac{1}{n}{\sum }_{i=1}^{n}\left|{y}_{i}-{\widehat{y}}_{i}\right|,$$

Mean Bias Error (MBE) defined as:

$$MBE=\frac{1}{n}{\sum }_{i=1}^{n}\left({y}_{i}-{\widehat{y}}_{i}\right),$$

and Root Mean Squared Error (RMSE) defined as:

$$RMSE=\sqrt{\frac{1}{n}\sum_{i=1}^{n}{\left({y}_{i}-{\widehat{y}}_{i}\right)}^{2},}$$

where *n* is the sample size, *y*_*i*_ is the estimated age and *ŷ*_*i*_ is the actual age*.* When validating the algorithms proposed by Stoyanova et al. (Sect. 4.1), our entire dataset is directly used as the test set. On the other hand, when evaluating the SASS and AANNESS methods, fivefold cross-validation is used. This means that the dataset is randomly partitioned into 5 equal sized disjointed subsamples, from which 4 are used to train the model and 1 is used to validate the model. This process is repeated until each of the 5 subsamples is used exactly once as the validation set.

### Ethics declarations

Ethics approval was granted by the Ethics Committee in Human Research of Khon Kaen University (Reference number HE601315).

## Results

For the whole sample of the 483 adult (18–92 years) *ossa coxae* of 374 males and females belonging to one Asian and four European identified osteological collections, we computed the accuracies according to the estimation models. These accuracies of individual models (presented in Table 3) are expressed by MAE, MBE and RMSE. Moreover, we decided to add two very naive models. The model denoted as “Random 18–92” estimates the age-at-death randomly within the interval of 18–92 years with uniform distribution. The results of MAE, MBE and RMSE for this model were 22.0, 0.8 and 27.4 years, respectively. The model denoted as “Constant 54.1” estimates the age-at-death as 54.1 years, which represents the mean age of all individuals. The values of MAE, MBE and RMSE for the Constant model were 14.1, 0.0 and 17.0 years, respectively. Although there is almost no age-at-death estimation power in these two models, they are useful for determining a baseline performance as a benchmark for other methods.

**Table 3:** Regression accuracy of various models.

Regression model	Entire dataset (18–92 years)		
	MAE	MBE	RMSE
Random 18–92^a^	22.0	0.8	27.4
Constant 54.1 (mean age)^a^	14.1	0.0	17.0
TPS/BE^b^	25.1	− 24.6	30.7
SAH^b^	21.4	− 20.2	27.2
VC^b^	16.2	− 6.6	20.6
VC + SAH^b^	19.2	− 16.8	24.8
VC + TPS/BE^b^	20.8	− 19.0	26.6
SASS (proposed #1)^c^	11.7	0.1	14.3
AANNESS (proposed #2)^c^	10.6	0.0	12.9

*MAE* mean absolute error, *MBE* mean bias error, *RMSE* root mean squared error, *TPS/BE* thin plate spline/bending energy, *SAH* slice and Algee-Hewitt score, *VC* ventral curvature, *SASS* Simple Automated Symphyseal Surface-based age estimation method, *AANNESS* Advanced Automated Neural Network-grounded Extended Symphyseal Surface-based age estimation method. When both right and left symphyses from a given individual are present, the estimated age is computed as the average from both bones. SASS and AANNESS are evaluated using fivefold cross-validation.

^a^Baseline models.

^b^Models by Stoyanova et al. ^25^.

^c^Proposed models by this study.

### Validation of algorithms proposed by Stoyanova et al.^25^

The results of the reached accuracies of the Stoyanova et al. models^25^ (denoted as “TPS/BE”, “SAH”, “VC”, “VC + SAH”, “VC + TPS/BE”) applied on our dataset are presented in Table 3. The MAE values ranged between 16.2 (VC) and 25.1 (TPS/BE) years where accuracy of the TPS/BE model was even worse than random estimation (22.0 years). The SAH model (MAE 21.4 years) was similarly unsuccessful. The values of MBE reached − 6.6 years at the minimum (VC) and 24.6 years at the maximum (TPS/BE) and shows strong underestimation of true age by the Stoyanova models. The results of RMSE were between 20.6 (VC) and 30.7 (TPS/BE) years, which is worse than or very close to random estimation (27.4 years).

### Results of the proposed computational approaches (SASS and AANNESS)

We proposed two approaches (SASS and AANNESS) for adult age-at-death estimation based on our dataset. The SASS method is based on multi-linear regression. The coefficients obtained for this model are presented in Table 4. These coefficients in combination with extracted features (Dirichlet normal energy, surface curvature, the total number of detected ellipses, the number of horizontally oriented detected ellipses, the number of vertically oriented detected ellipses, and the number of holes in the surface scan) allow for the age-at-death estimation when applied into the Eq. (1)—see section “Methods”.

**Table 4:** Computed coefficients for SASS method.

Coefficient	a_1_	a_2_	a_3_	a_4_	a_5_	a_6_	K
Value	3,118,517.53	191.36	0.10	1.03	− 3.18	0.14	17.85

a_1–6_ coefficients; K offset.

In the case of the SASS method, the MAE, MBE and RMSE values were 11.7, 0.1 and 14.3 years, respectively. For the AANNESS method, the results were 10.6, 0.0 and 12.9 years, respectively. The results are presented in Table 3.

### Validity of models and their relevance for age estimation

**Figure 8:**
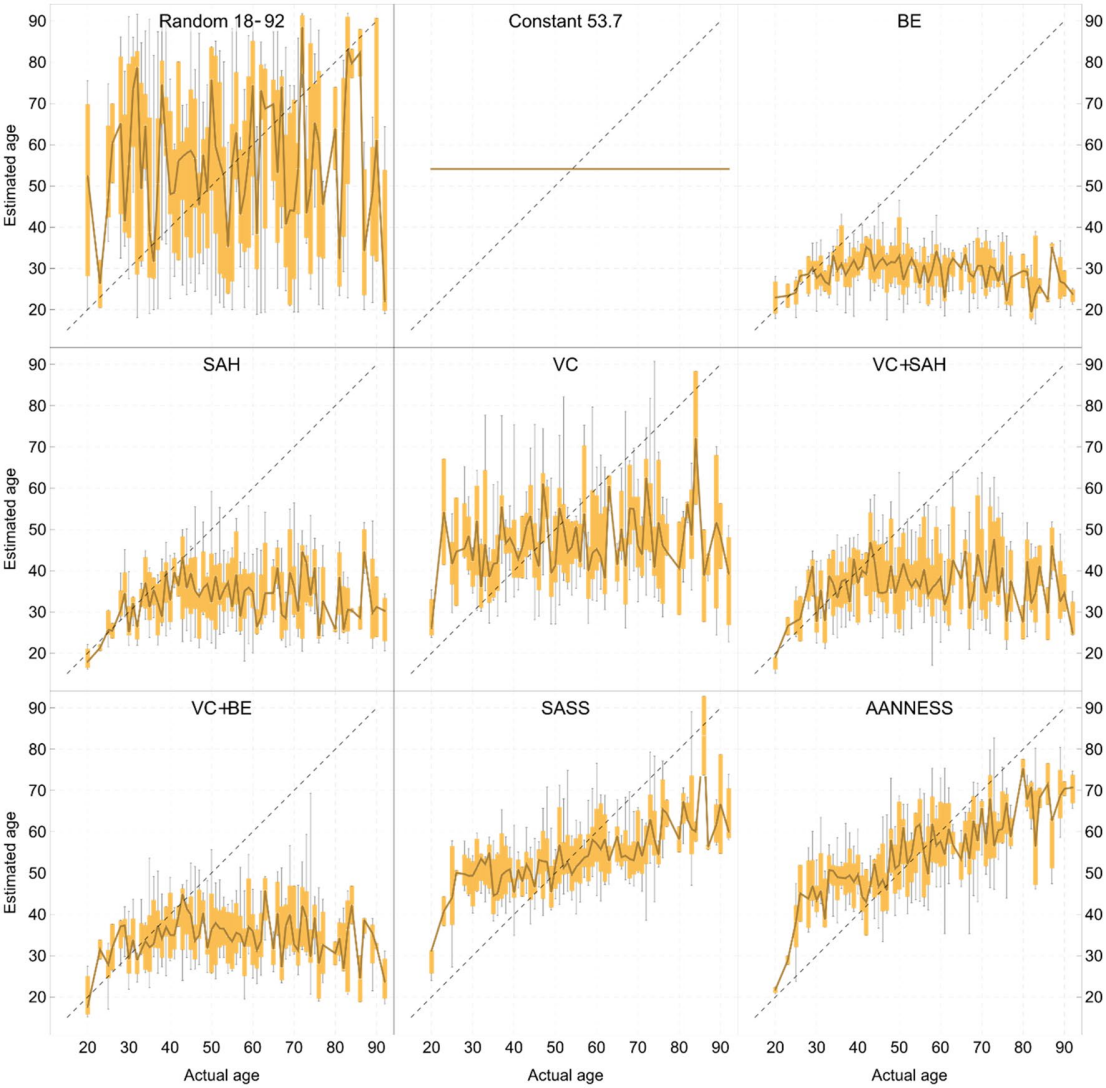
Visual comparison of all evaluated models from Table 4. Each image shows a variation of age estimations for a particular age for a given model. More specifically, for a certain age, the conventional box plot is used, i.e. it shows the minimum, maximum, q1, q2 (median) and q3 quartiles. The dark brown central line connects the median values. The black dashed line represents an ideal estimation, i.e. the closer to this line the model is, the better.

In order to show whether the compared models evince some systematic error, or whether certain age intervals show some anomalies in age prediction, we generated the graphs for each compared model (Random, Constant, original models of Stoyanova et al. and our two new models). They are presented in Fig. 8. Graphical representations show the variation of the age estimations per one-year age intervals for each age class. The conventional box plot is used to visualise the variation of age estimations for a particular age.

It could be seen that the models of Stoyanova^25^ applied in our dataset strongly underestimate the older individuals who are over approximately 50–60 years (with the exception of the VC model). We can interpret this to mean that these models are unable to estimate an individual's age above 50–60. Even though the models proposed in the present study, SASS and AANNESS, slightly overestimate the age of younger individuals and underestimate the age of older individuals, they are much closer to the ideal estimate (they are closer to the dashed line). This also demonstrates that both our models are able to estimate the age-at-death using the pubic symphysis across the entire adult age range, in contrast to our assumptions and observations of several researchers^2,8,43,44^.

## Discussion and perspectives

The pubic symphysis is by far the most employed skeletal indicator for age estimation of adults when it comes to non-destructive visual methods^2,31,45,46^. However, age estimation based on visual methods is still problematic, characterized by a strong subjectivity of evaluation and a high estimation error in the order of decades, especially with increasing age^5,47,48^. The symphysis, therefore, later became the subject of research for computational approaches^22,23,25,31,49^, as it is believed it reduces the subjectivity and potentially offers a new insight into aging patterns, especially those that are normally unseen by the human eye. Not only could the computational methods detect extremely detailed changes in the structures and automatically extract features, but these methods can also discover complex relations among particular features, which is almost impossible even for a human expert.

### Validation of existing algorithms (proposed by Stoyanova et al.^25^***)***

The most prominent computational approach for estimating adult age based on the analysis of surface changes in 3D models is the approach of Stoyanova et al. (2017)^25^. We applied their five models on our dataset and found significant discrepancies between the MAE (and RMSE and MBE as well) presented by Stoyanova et al. and the MAE computed on our dataset (e.g. 10.79 vs. 19.5 years for the VC + SAH model). As can be seen in Fig. 8, all the models by Stoyanova et al., except the VC model, very rarely estimate the age above 50–60 years (for our dataset). Even though the approach of Stoyanova et al. is relatively new, there are several validation studies on different population samples^27–29,50^. Kotěrová et al.^27^ conducted a validation on a pooled European sample and later on a Thai sample (unpublished^30^). Samples used in^27^ and^30^ are in fact small sub-samples of the significantly extended dataset used in this present study. Johnson and Bethard^29^ tested the Stoyanova models on a Portuguese sample, Joubert et al.^28^ on a sample of South Africans of European ancestry, and Figueroa-Soto et al. on skeletal samples from Latin America ^50^. Their results are quite consistent with those of the present study. In the original study, the accuracy of the estimated age (2 × RMSE value) was compared to the widely used method of Suchey and Brooks^2^. Stoyanova reached intervals that ranged from 27 to 32 years, which is more or less comparable to the error of Suchey and Brooks’ traditional method. However, in the present study, the interval for the models by Stoyanova et al. is 41–61 years. In our previous study^27^ based on the sample of 96 individuals with a mean age of 45 years, it was 36–44 years^27^. Slightly better results were found in the Thai population, with the age interval ranging from 30 to 40^30^. Johnson and Bethard^29^ reported very broad age intervals, ranging from 60 to 82 years. Joubert et al.^28^ also reported very low correlations with true age (RMSE values were not provided). Lastly, Figueroa-Soto et al.^50^ observed only a slightly larger magnitude of error (RMSE was also not provided). Figueroa-Soto et al. moreover proposed population-specific regression models; however, they found that they do not improve age estimates in their sample^50^.

We believe that such discrepancies between the results reported by Stoyanova et al. and those in this present study are primarily given by the unbalanced age distribution of the dataset used in Stoyanova et al. In fact, their dataset consists of 93 individuals, of which 48 (52%) are from the very narrow age interval of 16–39 years. Only 17 individuals (18%) from their dataset are older than 60 years. Apart from the unbalanced age distribution of the original Stoyanova sample, the size and composition of the test samples must also be taken into account. For example, Johnson and Bethard^29^ and Joubert et al.^28^ also used strongly unbalanced samples: they were under-represented by younger individuals. In terms of sample size, Figueroa-Soto et al. validated the method on a sample of 81 individuals. Lastly, Kotěrová et al.^27^ divided their dataset into samples of up to and including 40 years (n = 41) and those over 40 years (n = 55). Given that the second group (over 40 years) represents a wider age interval (20 and 42 years, respectively), the higher number of individuals in this subgroup is justified and convenient. The dataset used in the present study is almost uniformly distributed across all age intervals, as presented in Table 2, except the age interval of 18–29 years.

Another reason for such poor results when the models of Stoyanova were applied to our or other validation datasets could partially be seen in the population specificity^27,28,50^ and different aging rates between populations, since their models were proposed solely on American samples. Several studies reported population specificity when evaluating the performance of the traditional Suchey-Brooks method^48,51–53^, while in some others it was not demonstrated^54,55^. The population-specific models of Stoyanova’s computational method adapted for the Latin population did not improve the age estimates. The authors concluded that the current computational framework is an option for estimating age-at-death on their samples from Latin America^50^. However, to overcome the issue of population specificity and especially at this time, characterised by high levels of globalisation and migration^56–58^ resulting in the huge diversity of current populations, several researchers hold the opinion (including us) that it is necessary to use large samples consisting of several populations, such as in the following publications^6,8,13^, or more populations from eco-geographically close areas^59^. Such datasets cover much more variability of aging processes and could provide the basis for an accurate and reliable skeletal age estimation tool that is low in population sensitivity.

It should also be noted that the models of Stoyanova are restricted to male individuals only. Kotěrová et al., Kotěrová and Figueroa-Soto et al.^50^ also included male individuals only^27,30^, while Joubert et al.^28^, Johnson and Bethard^29^ and the present study incorporated both sexes. As the results of all the validation studies are similar, the inclusion of female individuals did not appear to affect the performance of the method. After all, Stoyanova et al. reported the successful application of their method to female individuals in a conference paper^60^.

Lastly, the comparability of 3D data acquisition and manipulation (isolation of the pubic symphyseal surface) also needs to be discussed as a possible source of error. Since different types of scanning devices and technologies (laser and structured light) have different resolutions and capture different levels of detail on the scanned surface (e.g. Structured Light Technology, which was used in this and our previous studies^27,30^, is known for providing higher resolution), it could be assumed that this will also affect subsequent analyses (e.g. results of the approach of Stoyanova et al.). This issue was already raised, for example, by Villa et al.^61^, Joubert et al.^28^ and in our previous study^62^ where different scanning technologies (laser vs. structured light) were compared. Among other things, we addressed this issue specifically for the method of Stoyanova and our results showed that various scanning technology does not affect age estimation, at least in the case of this particular method^62^. Another source of error, and therefore of differences in the performance of the age estimates, is the fact that 3D symphyseal surfaces have to be manually isolated before entering the analysis. As there are no clear descriptive instructions in the original study^24,25^ for the isolation of the symphyseal surface, Joubert et al.^28^ proposed a standardized protocol for this manual step. The authors of the Stoyanova method, however, confirmed strong repeatability and reproducibility among researchers during the isolation process^28,63^.

In summary, we see the following shortcomings in the Stoyanova et al. approach^25^, which we intended to overcome by proposing our own computational approach: (1) the models of Stoyanova et al. were designed for male individuals only, (2) they were computed in a small, unbalanced dataset from only one population, (3) the presented estimation errors are no better than those provided by traditional method errors, thus they are comparable.

### Advantage of the proposed computational approaches (SASS and AANNESS)

The main objective of the present study was to design our own model for estimating the age of adult individuals based on a 3D representation of the pubic symphyseal surface. The present article presents two models, SASS and AANNESS, which provide the deep analysis of a given 3D surface where the former extracts several features and uses multi-linear regression while the latter uses a neural network to estimate the age-at-death.

The proposed models are trained on a large multi-population dataset consisting of 483 bones coming from 374 adult individuals (both males and females). The size of the dataset and its composition (both sexes and different populations) allow for the more precise capturing of various age-related characteristics. The SASS model is designed in a way that makes it easy to interpret by researchers. The SASS model combines several features, each of which have certain age estimation capabilities. For instance, the number of holes in the scanned surface (i.e. the porosity) shows a certain correlation with the actual age-at-death of a given individual. The multi-linear regression takes the advantage of combining all such features together to make a more consistent age-at-death estimation, thus reducing the estimation error significantly. On the other hand, the AANNESS model relies on neural networks which are very hard to interpret by researchers. However, the AANNESS model extracts all the features automatically and provides better results compared to SASS. As the presented results (Table 4) indicate, our models outperform those that were developed by Stoyanova et al. in terms of all MAE, MBE and RMSE when applied on our dataset. When comparing the MAE, MBE and RMSE values computed in the original study^25^, the errors of our models may seem very similar. The MAE in their study (presented as inaccuracy) ranged between 10.8 and 12.9 years, while our models showed a MAE of 10.6 (AANNESS) and 11.7 (SASS) years. Stoyanova et al. reported the MBE (presented as a bias) from − 2.73 to − 1.82 years, which indicates a mild underestimation of the true age, while our models showed slightly more accurate values of MBE, i.e. 0.1 (SASS) and 0.0 years (AANNESS). Lastly, the RMSE of the Stoyanova models in their study were between 13.68 and 16.55 years, while our models in our study showed RMSE 12.9 and 14.3 years (AANNESS and SASS, respectively). However, as highlighted above, the fundamental differences regarding the input dataset (ours vs. the original study of Stoyanova et al.) must be kept in mind: that is, specifically, a significantly larger and more widely distributed dataset, consisting of both male and female individuals from various populations was used in this study.

Apart from the approach of Stoyanova et al., which offers three rather simple shape scores (bending energy, the SAH-Score and ventral curvature)^23–25^, there is the recent quantitative method of Bravo Morante et al.^31^. They draw attention to the limits of use, especially bending energy, which may result in high values in older individuals with advanced degeneration of the articular surface, thus underestimating their age. Therefore, they proposed a method based on bandpass filtering partial warp bending energy on the symphysis forms^31^ in order to improve the approach of Stoyanova et al.^25^. However, this paper has so far only been of an exploratory nature, as it is based on male individuals of one population only and does not provide a user-friendly application. The authors also conclude that the combination with other indicators may yield better age estimation results.

### Validity of the proposed models and their relevance for age estimation

The commonly accepted and used metrics^13,19,50^ of MAE, MBE and RMSE were used to evaluate the performance (regression accuracy) of all the tested and newly proposed models. All of them were used in the original study^25^, thus comparison with our results is easier. MAE (inaccuracy) measures the average magnitude of error without considering their direction, while MBE (bias) gives information about error direction, i.e. if the true age is under- or over-estimated. The RMSE gives a higher weight to large errors compared to MAE. This means the RMSE is preferred when large errors are particularly undesirable. Thus, RMSE is suitable for evaluating overall performance or for comparing the performance of different estimation methods, whereas MAE is more natural for researchers because of its linearity^64^.

Our models can estimate the age-at-death of an adult individual over the entire age interval (in our case between 19 and 92 years)—see Fig. 8. This contrasts with many authors^31,43,44,49,50,65^, according to which the pubic symphysis completes its age-related degenerative changes and then is not considered appropriate for age estimation of individuals over 40 or 50 years. In this respect, our results are unexpected. We believe this could be due to the use of the advanced computational approaches and data mining techniques applied on surface data. Mere visual assessment of skeletal indicators may not be sufficient to detect all the age-related surface changes. Nevertheless, more validation of SASS and AANNESS models in different samples is needed.

Even though our results suggest that the pubic symphysis may show age-related changes through the entire adult period, we advocate for the use of multiple skeletal indicators to estimate the age-at-death of adult skeletal remains. This is currently recommended as different skeletal indicators performed the best in the particular adult age range^43,49,66–68^.

Several new age-at-death techniques have been proposed in order to overcome the issue of subjectivity, which is one of the main imperfections of the traditional age estimation approaches regardless of the method used, with different levels of success^11,65,69^. The authors of a recent preliminary study^11^, for example, set out on the path of reducing subjectivity by means of the binary scoring of visually assessed signs on a pubic symphysis. The performance of the scoring system was evaluated by machine learning methods^11^. Based on this approach, the most reliable classification is possible when divided into three age intervals (≤ 29, 30–69, ≥ 70 years); furthermore, older individuals are more reliably classified using intervals of < 80 years and ≥ 80 years. The results of the present study, as well as some other computational studies, show that fully quantitative approaches that analyze three-dimensional surface data may be the right solution. They may considerably reduce or eliminate the subjectivity of the traditional gross morphological assessment, which is commonly associated with visual evaluation, and also reduce the requirements for the evaluator's experience^26,31,49,50,63^. They may also solve the low degree of standardization across application and practitioners^50^.

### Perspectives and future direction of research

Because quantitative computational methods have the potential to surpass traditional visual methods, due to the increased objectivity of evaluation and the possibility to detect age-related surface changes normally undetectable by the human eye, we will not limit our models to the surface of pubic symphysis. Other articulation surfaces such as the auricular surface of the ilium and the acetabulum should also be analyzed. The final output should be fully computational and should include more skeletal indicators, at the same time being user-friendly, i.e. an easy-to-use application.

## Conclusion

This paper presents validation results in a large dataset of a recent quantitative aging method ^25^ based on the pubic symphyseal surface. It was shown that the tested algorithms are not appropriate (MAE ranged between 19.2 and 25.1 years) for adult age estimation in our multi-populational sample containing both sexes. These results were considerably worse than the errors stated in the original study, which may be caused by several factors.

This study also aimed to develop computational approaches that would overcome some of the main drawbacks of current adult aging methods. As a result, two aging models using data mining and machine learning methods were proposed: SASS and AANNESS. These models produce very low errors (11.7 and 10.6 years, respectively) compared to traditional visual methods and were developed for both sexes in a multi-populational sample of individuals aged from 18 to 92 years. Surprisingly, our results also suggest that the symphyseal surface shows age-related changes across the entire adult age range, contrary to the findings of several researchers. A possible reason could be seen in the use of sophisticated data mining and machine learning tools in combination with 3D surface data.

For these reasons, the potential for further evolving our approaches is clear. Another intention is to include more skeletal indicators in one approach.

## Data Availability

The dataset included in this study is available from the corresponding author on reasonable request.
